# End of life care for infants, children and young people (ENHANCE): Protocol for a mixed methods evaluation of current practice in the United Kingdom [version 1; peer review: 2 approved]

**DOI:** 10.3310/nihropenres.13273.1

**Published:** 2022-05-13

**Authors:** Andrew Papworth, Julia Hackett, Bryony Beresford, Fliss Murtagh, Helen Weatherly, Sebastian Hinde, Andre Bedendo, Gabriella Walker, Jane Noyes, Sam Oddie, Chakrapani Vasudevan, Richard Feltbower, Bob Phillips, Richard Hain, Gayathri Subramanian, Andrew Haynes, Lorna K Fraser

**Affiliations:** 1 Department of Health Sciences, University of York, Heslington, York, YO10 5DD, UK; 2 Martin House Research Centre, University of York, Heslington, York, YO10 5DD, UK; 3 Social Policy Research Unit, University of York, Heslington, York, YO10 5DD, UK; 4 Hull York Medical School, University of Hull, Hull, HU6 7RX, UK; 5 Centre for Health Economics, University of York, Heslington, York, YO10 5DD, UK; 6 Parent Advisory Panel Member, York, UK; 7 School of Health Sciences, Bangor University, Fron Heulog, Bangor, LL57 2EF, UK; 8 Bradford Hospitals National Health Service Trust, Bradford, BD9 6RJ, UK; 9 Leeds Institute for Data Analytics, University of Leeds, Leeds, LS2 9NL, UK; 10 Centre for Reviews and Dissemination, University of York, UK, York, YO10 5DD, UK; 11 All-Wales Paediatric Palliative Care Network, Cardiff and Vale University Health Board, Cardiff, CF14 4XW, UK; 12 College of Human and Health Sciences, Swansea University, Swansea, SA2 8PP, UK; 13 Manchester University National Health Service Foundation Trust, Manchester, M13 9WL, UK

**Keywords:** paediatric, palliative care, end-of-life, mixed methods

## Abstract

**Background:**

Although child mortality has decreased over the last few decades, around 4,500 infants and children die in the UK every year, many of whom require palliative care. There is, however, little evidence on paediatric end-of-life care services. The current National Institute for Health and Care Excellence (NICE) guidance provides recommendations about what should be offered, but these are based on low quality evidence. The ENHANCE study aims to identify and investigate the different models of existing end-of-life care provision for infants, children, and young people in the UK, including an assessment of the outcomes and experiences for children and parents, and the cost implications to families and healthcare providers.

**Methods:**

This mixed methods study will use three linked workstreams and a cross-cutting health economics theme to examine end-of-life care models in three exemplar clinical settings: infant, children and young adult cancer services (PTCs), paediatric intensive care units (PICUs), and neonatal units (NNUs).

Workstream 1 (WS1) will survey current practice in each setting and will result in an outline of the different models of care used. WS2 is a qualitative comparison of the experiences of staff, parents and patients across the different models identified. WS3 is a quantitative assessment of the outcomes, resource use and costs across the different models identified.

**Discussion:**

Results from this study will contribute to an understanding of how end-of-life care can provide the greatest benefit for children at the end of their lives. It will also allow us to understand the likely benefits of additional funding in end-of-life care in terms of patient outcomes.

## Introduction

Although child mortality has decreased over the last few decades, around 4,500 infants and children (age 0–19 years) die in England and Wales every year^1,2^, and the number of children with life-limiting or life-threatening conditions has been rising. The latest figures estimate there are more than 86,000 children and young people with a life-limiting condition in England^3^. Approximately half of these deaths are from underlying life-limiting conditions^4^. Many of these children will receive end-of-life care, which is generally defined as support for people who are in the last years or months of their life^5^.

Over the last 30 years, there have been growing numbers of paediatric palliative care services in the UK that provide end-of-life care for children. Palliative care services for children and young people in the UK have developed locally with heavy reliance on individual clinicians and third-sector organisations (e.g. children’s hospices)^6^. We know that the way end-of-life care is managed and organised therefore varies considerably across the UK and between the different settings where end of life can occur^7^. End-of-life care for children has been described as ‘inconsistent and incoherent’^8^. Despite this, there is little evidence on the models of care, quality and resource implications and outcomes for children and families who use these services.

UK clinical guidance^9,10^ makes many recommendations such as the use of Advance Care Planning and pain management. However, the quality of evidence on which most of these recommendations are made is poor. Systematic reviews of specialist paediatric palliative care for children with cancer and other life-limiting conditions^11–13^ found that where medical specialists in palliative care are involved, children are cared for differently, with evidence of more advance care planning and less intensive care at the end of life. However, the conclusions that can be drawn are limited given the poor quality of the evidence and the reliance on North American studies, which are not necessarily transferable to the UK healthcare context.

A review of quality indicators to assess the impact of paediatric palliative care highlighted the breadth of indicators used, a lack of consensus, and limited input from the children’s perspectives^14^. Only a few studies have investigated families’ views or experience, or assessed how inequalities in access to care may influence end-of-life care^15–18^. The need for more research on end-of-life care has been identified in research prioritisation exercises^19,20^.

Furthermore, there has been just one economic evaluation of paediatric end-of-life services^8^, and this highlighted the poor current level of understanding about the costs of care^21^. Previous research^8,22,23^ and the UK’s NICE Guideline^9^ has emphasised the challenges of conducting economic evaluation in end-of-life care, as conventional health maximisation is no longer the aim of the intervention. Increasingly, economic evaluations of health care interventions are used to inform decisions on how best to allocate limited resources for optimal health gain. In end-of-life care, however, a comprehensive view of the costs and benefits that are relevant extends beyond health to encompass broader cross-sector impacts spanning the statutory and non-statutory sectors, as well as the private sphere, to encompass the impact on the patient and their network of family and friends.

In addition, dimensions of care beyond health are important since patient care is no longer primarily curative, nor with any likely extension in time lived. This makes economic evaluation of palliative care non-standard^8,9,22,24,25^. Failure to consider the costs or benefits of the range of end-of-life care packages has contributed to the inconsistent and variable provision of care throughout the NHS, and internationally. Furthermore, at a time of extensive budgetary pressures, the inability to define the benefits of a healthcare budget or argue for the value of additional funding has led to increasing reliance on third sector support—a sector which itself is under substantial pressure^26^.

Therefore, this study aims to identify and compare different models of providing end-of-life care for infants, children and young people in the UK, in terms of outcomes and experiences for children and parents, resource use, and costs to families.

For the purposes of this study, we are defining end-of-life care as that which is delivered when the health professionals caring for a child would not be surprised if the child did not survive the next 12 months.

This protocol adheres to the SPIRIT checklist for reporting clinical trial protocols^27,28^.

## Methods/design

The study has five objectives, which are referred to throughout this protocol paper:

1. To identify and describe current models of delivering end-of-life care to infants, children, and young people (0–25 years) in the UK.
2. To identify barriers and facilitators to the implementation of these end-of-life care models.
3. To assess inequalities in access or availability of these models.
4. To explore whether and how the outcomes and experiences of infants, children, young people, and families vary dependent on the model of end-of-life care received.
5. To compare the resource implications of the different models of end-of-life care for the health providers and families.

**Figure 1:**
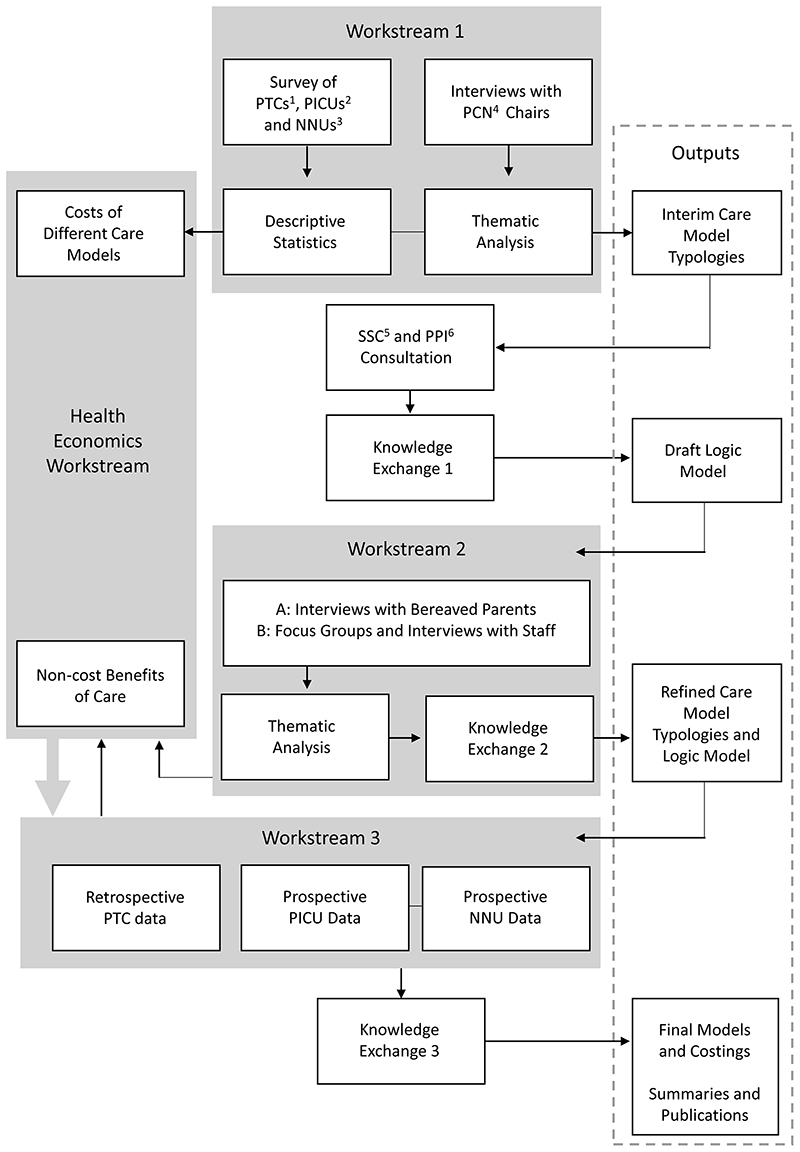
Schematic of the ENHANCE project. Abbreviations: 1 Children and Teenage and Young Adult (TYA) Cancer services – Principal Treatment Centres 2 Paediatric Intensive Care Units 3 Neonatal Units 4 Palliative Care Network 5 Study Steering Committee 6 Patient and Public Involvement.

To achieve these objectives, this study will use a multistage mixed methods framework^29^ with three linked workstreams (WSs) and a cross-cutting health economics theme (see Figure 1). It is focused on three clinical settings that together care for approximately 50% of the children who die in the UK each year:

1. Children and Teenage and Young Adult (TYA) Cancer Services – Principal Treatment Centres (PTCs) (~350 deaths per year)^30^
2. Paediatric Intensive Care Units (PICUs) (~700 deaths per year)^31^
3. Neonatal Units (NNU) i.e. Special Care Baby Units (SCBU), Local Neonatal Units (LNU), Neonatal Intensive Care Units (NICU) (~1100 deaths per year)^32^

### Ethics approval and consent to participate

WS1 has been approved by the Department of Health Sciences Research Governance Committee, University of York (HSRGC/2020/418/G). WS2 has been approved by the NHS REC and HRA (21/WS0170) (IRAS ID: 300913). WS3 part 1 has been approved by the NHS Health Research Authority (REC reference: 21/NW/0009; CAG reference: 21/CAG/0026). Applications will be submitted to the HRA for WS3 part 2. All participants will be required to give written informed consent.

### Workstream 1 (WS1)

WS1 will identify and describe current models of providing end-of-life care in UK NNUs, PICUs and PTCs (objectives 1, 2, 3 and 5).

#### Design

An online survey of clinical, or palliative care leads of all PICUs, NNUs and PTCs in the UK and semistructured interviews with the Chairs of the regional children’s Palliative Care Networks (PCNs), which bring together regional stakeholders to improve care.

The survey content will be informed by existing evidence and clinical guidance on factors identified as relevant to end-of-life outcomes and experience^9,33–36^, and through consultation with the study’s Parent Advisory Panel (PAP) (see Patient and Public Involvement (PPI) section).

The survey will collect data on the following: how each unit is organised and staffed (e.g. type of hospital, professions represented in the multi-disciplinary team); annual ‘caseload’ and number, and places of deaths; access to, and use of, medical and nursing neonatal or paediatric palliative care expertise; availability of, and ways of working with, community services (e.g. children’s community nursing team, children’s hospice); within-service practices and policies regarding planning for end of life; bereavement support; unit/ward layout and the availability of dedicated spaces for end-of-life and bereavement care.

The final draft survey will be piloted with at least one palliative care/clinical lead from each type of setting (PICU, NNU, PTC). Cognitive interviewing techniques^37^ will be used to evaluate content, feasibility, respondent burden, and the wording of question and response options.

Additional information on service costs will be collected from operational/business leads of a sub-sample of services represented in the survey respondents. Where a survey respondent reports their hospital has a consultant-led paediatric palliative care service, we will contact the service for further information on the make-up of that service, and its funding and commissioning arrangements. These data will be collected via an online survey or a structured interview.

The semi-structured interviews with the Chairs of all the UK regional children’s PCNs will gather information on: NHS and third sector paediatric palliative care services and professionals in the region; views on equity of access to paediatric palliative care at end of life in terms of geographical location, age and diagnosis; the ways the network supports service development and equity of access to high quality end-of-life care; and the barriers to achieving this.

#### Sampling

Palliative care or clinical leads/directors of all UK NNUs, PICUs and PTCs will be invited to complete the survey, or to cascade completion to the unit/centre’s palliative care lead. Chairs of all UK regional PCNs (n=16) will be invited to participate in an interview.

#### Recruitment

Professional member organisations and networks will distribute an email invitation to take part in the survey on behalf of the research team, and we will also advertise the study via social media. The survey will include the participant information sheet (PIS). The identity and contact details of Chairs of regional PCNs is publicly available. They will be invited to take part via an email from the study team, which will also have a copy of the PIS attached.

#### Data collection

The survey will be hosted on the Qualtrics© survey 7 platform^38^ (RRID: SCR_016728) (An open-access alternative that can provide an equivalent function is Survey Monkey). Email reminders will be used to maximise response rates. The interviews will be conducted via telephone (audiorecorded and transcribed) or video call (recorded).

#### Data analyses

The objective of the analysis of the survey data is to develop a typology of current approaches/service models of delivering end-of-life care, and provide a descriptive account of these models and their occurrence/distribution across NNUs, PICUs, and children’s and TYA PTCs.

Data will be extracted into R^39^,^40^ (RRID: SCR_001905), from Qualtrics©. We will use Upset Plots (software package ComplexUpset v1.3.3)^41,42^ to visualise patterns in the way service characteristics identified *a priori* (see Design above) are relevant to end-of-life outcomes and experiences^35,36,43^ (see Design above).

Once identified, cross-tabulation will be used to compare end-of-life care delivery models with respect to organisational, patient, and contextual characteristics and region. A thematic analysis^44^ of interviews with regional PCN Chairs will be used to understand and compare views on end-of-life care across regions, and the barriers to, and facilitators of high quality end-of-life care.

#### Integration with other workstreams

An initial logic model representing end-of-life care models will be developed by drawing on the findings of WS1 and existing literature^14,33,34,45–48^. These will inform plans for the subsequent workstreams, e.g. topics guides and participants for WS2, and variables and outcomes for WS3.

Preliminary WS1 findings will be presented at a sector knowledge exchange event, with these discussions informing the final stages of work specifying alternative delivery models, and the selection of research sites for WS2. A paper reporting findings will be submitted to a peer-reviewed publication.

### Workstream 2 (WS2)

WS2 will evaluate and explore how the delivery and experience of end-of-life care provision varies according to the different models identified in WS1 (objectives 2, 3, 4 and 5).

#### Design

WS2 of ENHANCE will use qualitative methods and draw upon the principles of thematic Framework Analysis^27^, which will enable comparisons of similarities and differences in parents’ and professionals’ accounts to be made across each model.

Data will be drawn from interviews with bereaved parents and from focus groups with health professionals. The interviews will explore parents’ experiences of their child’s treatment and care towards the end of their life, as well as perceived outcomes and costs to them. The focus groups will explore health professionals’ experiences of meeting end-of-life care needs and factors affecting access and delivery, including identification of any inequalities. These may be supplemented with individual interviews with other service staff (e.g. business managers) to explore resource implications of the different models.

#### Sampling

PTCs, PICUs, NNUs will be purposively sampled to ensure representation of those that best fit each of the models identified in WS1. If, for example, six models are identified in WS1, parents and health professionals in approximately three services per model will be sought.

A minimum of seven parents (matching the inclusion criteria in Table 1A) per end-of-life care model will be interviewed (e.g. if six models are identified, total n≥42 parents), with care taken to ensure relevant clinical and demographic characteristics are represented, including: child age and their role in decision making; underlying diagnosis; expected/unexpected death; place of death; family composition; ethnicity; and socioeconomic status. This diversity will be achieved using two strategies: 1) screening of children’s records in advance of recruitment; and 2) monitoring of data collected in order to focus later recruitment on characteristics not reflected in the initial sample.

Parents whose children died in the last three months or more than three years ago (at the time of recruitment) will be excluded^49,50^. This decision has been made with reference to relevant research^49,50^, our own experience of conducting research with bereaved parents^51^, and input from the study PAP.

A minimum of three health professional focus groups per end-of-life care model will be conducted (e.g. where six models are identified, there will be 18 focus groups in total with each attended by at least six participants, total n>108). The inclusion criteria for health professionals is outlined in Table 1B.

Final sample sizes will be determined by the number of end-of-life care models identified in WS1 (and therefore explored), and the final sampling criteria for WS2, which will be informed by WS1 findings, existing literature, and input from the study PAP.

#### Recruitment and consent

Parents will be recruited in one of two ways: a) identified by clinical teams in the selected sites; b) recruited by social media, noticeboards and newsletters.

In the first instance, clinical teams will review children’s records to identify eligible parents and will make first contact. Interested parents will be asked to complete a ‘consent-to-contact’ form and return it to the research team, who will then contact them directly to explain the study and arrange an interview. In the second instance, the study will be advertised via social media, posters and leaflets where appropriate, and parents will contact the research team directly.

Parents will be asked to indicate a trusted health professional that they would like the research team to contact in case they become distressed during, or have any concerns about, the interview. During interviews, the researcher will monitor (observation of verbal and nonverbal behaviours) and proactively re-check consent. At the close of the interview, consent will be re-checked and all parents will be offered a follow-up call from the study team.

For the focus groups, the local Principal Investigator (PI) at each participating NHS site will identify suitable participants, circulate recruitment materials, and coordinate arrangements. Interested staff will be asked to contact the research team directly. Following the focus groups, the study will decide whether additional data on end-of-life care costs and resources are needed, and will liaise with the local PI to identify suitable participants and invite them to take part in an interview.

All potential participants (parents and health professionals) will receive a PIS explaining the study and what will happen to them and their information if they take part, and all participants will provide written or electronic consent in advance of taking part in an interview or focus group.

#### Data collection

The parent interviews, which will take place face-to-face or via video or telephone call, will be split into a narrative section (inviting parents to tell their story of their child’s end of life and the period immediately following) and a section using a semi-structured topic guide. This has the benefit of allowing parents to share their experiences without imposing a structure or order in the first part of the interview but ensuring that key topics are consistently covered with all participants. The topic guide for the latter section will be informed by: WS1 findings; consultation with key stakeholders; and existing research that has explored end-of-life care provision with families^17,47,51–56^. It will also be piloted with at least two parent advisors. The topic guide will include an exploration of important outcomes associated with end-of-life care and resource implications for families (e.g. costs of car parking, time off work), to inform WS3 and the cross-cutting health economics theme.

The focus groups (lasting ~90mins) will be located on Trust premises or conducted via video call (e.g. Microsoft Teams, Zoom) to facilitate attendance of staff working in different services and organisations. Each focus group will consist of 6–12 staff participants working in different services operating the same end-of-life care model. Thus, a single focus group will include 2–3 staff from each of the three main services (i.e. PTCs, PICUs, NNUs), and also where relevant 2–3 staff from services feeding into the end-of-life care model (e.g. children’s hospice, children’s community nursing team). Each focus group will also include staff working in different roles (e.g. physicians, nurses, allied health professionals).

A topic guide will be used to structure the focus groups, and will be informed by WS1 findings, stakeholder consultation and existing research^57^. As with the parent interviews, the topic guide will include exploration of important outcomes associated with end-of-life care and resource use.

Interviews and focus groups will be conducted by experienced qualitative researchers. Face-to-face and telephone interviews will be audio-recorded using an encrypted digital audio recorder, and video calls will be audio-recorded by the video conference platform. All interviews and focus groups will be transcribed (intelligent verbatim) for analysis by an external, GDPR-compliant transcription company with experience of transcribing data collected for health research.

Researchers involved in data collection will also keep field notes throughout the data collection process, commenting on important non-verbal data and interesting observations to either follow-up in subsequent interviews or focus groups, or to explore during the analysis process.

#### Data analyses

The data for analysis will comprise interview and focus group transcripts, audio-recordings, and the researchers’ field notes. The data will be analysed using thematic Framework Analysis (in a six-step process, see Table 2) to draw out key themes that “capture something important about the data in relation to the study objectives, and represent some level of patterned response or meaning within the data set”^39^. Data will be analysed by model to facilitate an understanding of similarities and differences between the models. Parent and health professionals’ data for each model will be analysed together and compared during the development of themes to identify similarities, differences, and disagreements.

Up to five analysts will work together through the analytical steps. One of the analysts will be the study’s parent co-investigator (see PPI section), who will receive appropriate training and support for the role. Having up to five analysts working together and including a parent as analyst will help to ensure rigour, authenticity and dependability of findings^40^. The wider research team and study PAP will be utilised at key points during the analysis to help identify and refine the key themes that represent the data and interpret their meaning.

#### Integration with other workstreams

The results of this workstream will help refine the logic model for end-of-life care for children and contribute towards an understanding of factors affecting access to, and uptake of end-of-life care across the models. Model typologies will also be refined and expanded. Outputs will be presented at a second knowledge exchange workshop (month 34) for discussion and to aid our interpretation of the findings. The final results will be presented in a peer-reviewed publication.

### Workstream 3

WS3 will evaluate and explore the impacts of the different models of end-of-life care provision on child and parent outcomes (objectives 2-5). Using routinely collected data for children with cancer and prospective data collection in PICUs and NNUs, WS3 will assess whether outcomes vary according to the different models of end-of-life care.

#### Part 1 – Cancer PTCs, PICUs and adult ICUs

##### Design

This part of WS3 will perform retrospective secondary analysis of routinely collected data of linked population-level datasets available for children, teenagers and young adults (up to age 25 years) with cancer and who died from 2012–2020 in England (n≈4000). We will assess whether the use of ‘high intensity’ treatments (see data analysis section below) in infants, children and young people who have died from cancer varies depending on the model of end-of-life care that their service delivered.

##### Data collection

The data sources, variables and process of data linkage are summarised in Table 3 and the variables are summarised in Table 4. After linkage, pseudonymised data will be securely transferred to the University of York for data analyses.

##### Data analyses

We will use R^39,40^ to analyse the data for this portion of the study. After linkage has concluded, an assessment of data quality and completeness will be undertaken for all the key clinical and demographic variables of interest. An assessment of missing data will be undertaken once the data are linked and multiple imputation using chained equations will be used where appropriate^58^. If imputed datasets are used, sensitivity analyses comparing complete case analyses with the imputed analyses will be undertaken.

Some key demographic variables (e.g. ethnic group, deprivation score) will be obtained by combining different data sources. If any conflict between data sources occurs, we will assign the most commonly recorded ethnic group (census 2011 categories) assuming that it is not ‘unknown’.

Appropriate summary statistics, e.g. frequencies and proportions for categorical variables and mean (with standard deviation) or median (with interquartile range) for continuous variables will be produced for all the key variables to describe any variation.

The current definition of ‘high-intensity care’ at the end of life was developed from research in adult palliative/end-of-life care in North America (listed as high intensity treatments in Table 5). This definition has been used in paediatric end-of-life research in Canada and the US. In order to make the definition more culturally appropriate, we hosted a virtual (via Zoom) consultation event with paediatric oncology and haematology experts.

Analyses will evaluate and compare outcomes used in different end-of-life care models (identified in WS1) using appropriate regression models. Each analysis will account for the multiple confounding factors in this population (age, underlying diagnoses, comorbidities, outpatient attendance, socioeconomic status (Index of multiple deprivation))^59^ identified using causal inference methods^60–62^.

#### Part 2 – PICU and NNU

##### Design

This part of WS3 will use prospective longitudinal data collection from clinicians and parents to explore additional individual level outcomes beyond those in Part 1. PICUs (approx. 10–12 units) and NNUs (approx. 40–50 units) in the UK will be purposively sampled to include examples of each model identified in WS1, as well as other factors including size, geography and distance from key end-of-life care providers.

##### Data collection

Prospective data collection using BadgerNet (NNU) and PICANet (PICU), including quality indicators of care^63^ and outcomes up to and including death, with these informed by WS1 and WS2.

A deferred model of consent will be used: clinical teams will record standardised information on children’s outcomes prior to death and parents will then be approached to consent to the study if their child dies^64^. Previous studies have shown how difficult it is to obtain consent in the PICU setting^65^, there is debate over whether true informed consent can be obtained at times of very high levels of anxiety^66^ and in the intensive care setting^67,68^, and there is evidence that deferred consent is acceptable to parents in this context^69,70^. The research team will not receive any data until parents have consented to inclusion in this study. Data will be collected for ~1200 children (to yield 800 deaths) over an 18-month period.

PICUs: Children will be identified by PICU staff (in approx. 10–12 PICUs) when they are at high risk of death (e.g. starting to discuss do not attempt resuscitation (DNACPR)). We will need to recruit ~600 children to capture ~400 deaths.

NNUs: Infants will be identified by NNU staff (in approx. 40–50 NNUs) at the point that they are identified at risk of death (e.g. high clinical risk index for babies (CRIB) score^65^), severe hypoxic-ischemic encephalopathy^66^, extreme prematurity (23/24wks) or starting to discuss DNACPR (we will need to recruit ~600 babies to capture ~ 400 deaths).

If there are six models of care to explore and compare then with a sample size of 800 we would have 80% power to detect differences of the magnitude of 0.44 (i.e. effect size) on the primary outcome quality of death scale^67^. If there are fewer models of care then smaller differences could be detected whilst retaining 80% power (e.g. 0.34 with four models of care).

Utilising current clinical IT platforms (BadgerNet for NNU and PICANet^31^ for PICU) we will collect information on quality indicators of care^36^ and outcomes up to and including death. These outcomes will be informed by WS1 and WS2, but will likely include symptoms, choices offered to parents about place of care, involvement of SPPC team, place of death, presence of an advance care plan, and bereavement support offered. These data will be collected prospectively by the clinical team, with additional data collection from the parents, via postal, online or telephone questionnaire approximately three to six months after the child’s death. These data will include: a quality-of-death scale to assess end-of-life care; one of the tools for economic evaluation (ICE-CAP-CPM^68^, PICU-QODD-20^68,69^ or the children’s palliative care outcome scale (cPOS)); an assessment of parent outcomes using the EQ-5D-5L^70^; and data to explore the resource use and cost implications beyond secondary care, including primary care, hospice care, and parental out of pocket costs and reduction in, or loss of employment. We will determine the most appropriate tool to use to assess end-of-life care based on the findings of WS1 and WS2, and through consultation work with our Parent Advisory Group.

##### Data analyses

We will use R^39,40^ to analyse the data for this portion of the study. Clinical and demographic data of the infants and children who have died and their parents will be summarised in a table using descriptive statistics. Continuous measures will be reported as means and standard deviations or medians and interquartile ranges (as appropriate), and categorical data will be reported as counts and percentages. The flow of participants through the study will be presented in a diagram detailing reasons for withdrawal where data are available.

The quality-of-death scale chosen to assess end-of-life care will be analysed using multiple linear regression with the quality-of-death scale as the outcome and model of end-of-life care as the independent variable of interest adjusting for the multiple confounding factors in this population (e.g. age, underlying diagnoses, co-morbidities).

Model assumptions will be checked and if they are in doubt the data will be transformed prior to analysis. The difference between the different models of care in the mean quality-of-death scale and corresponding 95% confidence interval will also be presented. Other outcomes of interest will be analysed using an analogous approach to that outlined above for the quality-of-death scale.

##### Integration of WS3 with other workstreams

Findings from WS3, and the other final outputs, including the statistical model typologies, will be presented as part of the third knowledge exchange event in month 48 and will be presented in a peer-reviewed publication.

### Health economics cross-cutting theme

The health economics component of the study is a cross-cutting theme. It will identify and estimate the relevant outcomes and resource-use implications of the provision, or lack of end-of-life services, with an underlying aim of considering how such services can be meaningfully assessed using economic evaluation frameworks of cost-effectiveness to inform future value assessments of end-of-life services in these settings. This component will be embedded within the three workstreams. It will contribute to the planned summaries of the cost of the different models of care in addition to informing the structure of subsequent resource-use explorations in WS2 and WS3.

#### Design and data collection

Objective 4 (the outcomes associated with the models of care) will be assessed primarily through collaboration with the novel data collection in WS2 and WS3. These will be used to inform a summary of the non-cost benefits associated with each care model and an assessment of whether outcomes for children and families vary according to the different models of end-of-life care.

Objective 5 (the cost of the different models of end-of-life care) will be explored in several ways, including a review of the available published and grey literature, use of survey and discussion-based elicitation methods as part of WS1 and WS2 respectively, and regression analyses of the resource use associated with the different models of care alongside WS3 Part 1 (secondary analysis of retrospective data from across the three clinical settings).

#### Output

The findings from these activities will be used to inform an analysis of the costs of providing the services and who bears them, alongside an exploration of the variation in the cost of the end-of-life care models through extensive sensitivity and scenario analyses. The cost and outcome estimates from these objectives will, if appropriate, be combined to inform a cost-consequence analysis of the different models of end-of-life care in the respective populations. The health economics component will also consider the appropriateness of existing cost-effectiveness evaluation frameworks to the clinical setting.

### Synthesis of workstreams

After WS3, the care model typologies and logic model will again be expanded to incorporate the new findings. Further interpretation of findings will be achieved through a study team workshop followed by the final knowledge exchange workshop focused on: i) what the common and distinct features (including outcomes, barriers, enablers, experiences, delivery aspects etc.) are across the models; and ii) developing conclusions on the study’s overall implications for policy, practice, research.

### Patient and public involvement (PPI)

The study’s PPI plans have been designed with reference to the NIHR INVOLVE National Standards for Public Involvement^72^. A study-specific Parent Advisory Panel (PAP) (comprising around 10 parents with different experiences of end-of-life care) has been established and will be involved at all stages of the study. The group will meet two to four times each year, depending on the involvement required at different stages in the study. Other planned activities where closer involvement/input is required will be undertaken by one or two members, with appropriate training where required. Specific input is outlined in Table 6, below.

The study’s parent co-applicant will work alongside the study team, represent the PAP at management team meetings and SSC meetings, attend PAP meetings, provide support to other parent members throughout the study, be involved in data analyses in WS2, and assist with dissemination of study findings. A PPI log, guided by the Public Involvement Impact Assessment Framework^73^, will record planned and unplanned involvement, and how this involvement impacts on the study.

### Study status

The data collection for WS1 has been completed and data analysis is currently ongoing. We have started to recruit NHS sites for WS2. We have submitted data access requests for WS3a.

## Discussion

Results from this study will identify and compare models of end-of-life care for infants, children and young people in terms of child and parent outcomes and their resource implications. It will allow researchers, decision makers, and the children and their families to contribute to an understanding of how we can ensure that the limited funding for end-of-life care can be used for greatest benefit for children at the end of their lives. It will also outline the likely benefits of additional funding in end-of-life care in terms of outcomes for children and their families.

Ensuring that the results of our study are incorporated into updated versions of clinical guidelines and policy statements is important. This will be achieved through directly informing the key professional organisations, including NHS England and NICE, and also through publishing the findings in peer-review journals. There may be some resistance to change within organisations, but having the key professional organisations engaged with this study throughout should enable more effective implementation of these study findings.

## List of abbreviations

cPOS: Children’s Palliative Care Outcome Scale
CRIB: Clinical Risk Index for Babies
DNACPR: Do Not Attempt Cardiopulmonary Resuscitation
HSRGC: Health Sciences Research Governance Committee (University of York)
HES: Hospital Episode Statistics
ICNARC: Intensive Care National Audit and Research Centre
ICU: Intensive Care Unit
LNU: Local Neonatal Unit
NCRAS: National Cancer Registration and Analysis Service
NHS: National Health Service
NICE: National Institute for Health and Care Excellence
NICU: Neonatal Intensive Care Unit
NIHR: National Institute for Health Research
NNU: Neonatal Unit
ONS: Office for National Statistics
PAP: Parent Advisory Panel
PCN: Palliative Care Network
PHE: Public Health England
PI: Principal Investigator
PICANet: Paediatric Intensive Care Audit Network
PICU: Paediatric Intensive Care Unit
PIS: Participant Information Sheet
PPI: Patient and Public Involvement
PTC: Principal Treatment Centre
RTDS: Radiotherapy Dataset
SCBU: Special Care Baby Unit
SPPC: Specialist Paediatric Palliative Care
TYA: Teenage and Young Adult
WS: Work Stream

## Figures and Tables

**Table 1:** Inclusion criteria for WS2 participants.

**A: Parents**	
1	Bereaved parents/guardians where the deceased child was between neonatal to 25 years old at the time of death
2	Parents/guardians whose child has died in the last three years, and no sooner than in the last three months
3	Bereaved parents/guardians whose child was treated in one of the study’s three settings (PTCs, PICUs, NNUs)
4	Bereaved parents/guardians who are able to speak and understand English
5	Bereaved parents/guardians who are able to give written and informed consent
**B: Health professionals**	
1	Staff who work in one of the study’s three settings (PTCs, PICUs, NNUs), or for a service that is identified as being important for the functioning of one of the end-of-life models identified in WS1
2	Staff who have worked with children who have died between neonatal to 25 years old
3	Staff who are able to speak and understand English
4	Staff who are able to give written and informed consent

**Table 2:** Six steps of thematic framework analysis to be applied to WS2.

Stage	Description
1: Familiarisation with the data	All the analysts will read and re-read all the transcripts — parent transcripts first and then staff focus group data second — to explore how similar they are in content.
2: Generating initial codes	One analyst (A1) will make notes of interesting concepts and ideas (referred to as ‘codes’ from hereon) that relate to the research objectives. The other analysts will read a proportion of transcripts for each model and note down commonly occurring codes. Working together, the analysts will discuss the selection, labelling and meaning of codes. At this stage, the data will be managed and coded in NVivo software (RRID: SCR_014802)^41^.
3: Developing a working analytical framework	A1 will continue to generate codes that represent the data and discuss these regularly with the other analysts. All researchers will meet and agree on a set of codes to apply to all subsequent transcripts. The analysts will work together to identify categories that represent the data and explore relationships between codes and categories, e.g., identifying how groups of codes may be combined to generate categories, and how these relate to the different models of end-of-life care. Similarities and differences between the models will be explored during this step. A working analytical framework will be created, possibly through a process involving several iterations.
4: Applying the analytical framework	All analysts will then work together to apply the working analytical framework to all subsequent transcripts.
5: Charting data into the framework matrix	All analysts use a spreadsheet document to generate a matrix with cases (participants) along the rows, and categories placed along the columns. Into this matrix, the data from the interviews and focus groups will be ‘charted’ or inputted. This will require a balance between summarising the data so it is manageable whilst still retaining the original meanings and context of the original data.
6: Interpreting the data	During all these stages, the analysts will work with the wider research team and the PAP to review and refine the categories and the analytical framework. Each final category will be defined and described using quotations to illustrate meaning, and the study findings will be incorporated into the model typologies and logic model developed in WS1, e.g., adding descriptive details about implementation, causal mechanisms, outcomes.

Adapted from Guest *et al.,* 2012^44^

**Table 3:** Data sources, WS3, Part 1: Children in Cancer PTCs, paediatric and adult ICUs.

**Data Sources**
Paediatric Intensive Care Audit Network (PICANet)
ICU Case mix Programme (ICNARC)
Public Health England (PHE) National Cancer Registration and Analysis Service (NCRAS)
Hospital Episode Statistic Data (HES) for admitted care, outpatient, and A&E
Systemic Anti-Cancer Therapy Data set (SACT), i.e. chemotherapy.
Radiotherapy Data Set (RTDS)
Office for National Statistics (ONS) death certificate data
**Data Linkage**
The data sources held by PHE are already linked on an individual level
The PICANet and ICNARC data will be linked by PHE using deterministic data linkage techniques using dataset serial number, NHS number, name, date of birth, sex, and postcode.

**Table 4:** Key variables / source data, WS3, Part 1: Children in Cancer PTCs, paediatric /adult ICUs.

NCRAS (Primary dataset):	Treatment Data (SACT/RDTS)	Intensive care data (PICANet data)	Hospital admission data (HES)	Outpatient data (HES)	A & E data (HES)	Death registration data (ONS)
Cancer diagnoses Age Sex Date of Diagnoses	Chemotherapy and dates Radiotherapy and dates	Age Sex Ethnicity Deprivation score Planned or unplanned admission Date & time of admission Source of admission Date of discharge Destination on discharge Date of death (if occurred) Primary reason for admission Comorbidities Paediatric Index of Mortality score and variables used to derive this Daily intervention data (e.g.mechanical ventilation, inotropic support, renal replacement therapy)	Age Sex Ethnicity Deprivation score Diagnoses (ICD10 codes) Procedures (OPCS codes) Date of admission Source of admission Specialty of admission Emergency or planned admission Date of discharge Discharge destination Date of death (if occurred)	Age Sex Ethnicity Deprivation score Date of appointment Specialty of appointment	Age Sex Ethnicity Deprivation score Date and time of attendance Diagnoses/reason for attendance Outcome Treatment	Date of death Cause(s) of death Place of death

**Table 5:** Key outcomes for WS3, Part 1: Children in Cancer Principal Treatment Centres.

**Primary outcome**
Any one of the following high intensity treatments: intravenous chemotherapy <14 days from death (yes/no); more than one emergency department visit (yes/no); and more than one hospitalization or intensive care unit admission <30 days from death (yes/no)^71^.
**Secondary outcomes**
Mechanical ventilation <14 days from death, place of death (hospital, home, hospice).

**Table 6:** Input from the ENHANCE Parent Advisory Panel.

Workstream	Tasks
WS1	Key characteristics to be included in the survey Selection of models
WS2	Review of consent materials and recruitment process Topic guide development Analysis
WS3	Selection of outcomes and measures Review of consent materials and recruitment process
General	Integration following each workstream (e.g. developing model typologies and logic model) Planning and attending knowledge exchange events

## Data Availability

No data are associated with this article. Zenodo: SPIRIT checklist for ‘End of life care for infants, children and young people (ENHANCE): Protocol for a mixed methods evaluation of current practice in the United Kingdom’. https://doi.org/10.5281/zenodo.6420928^28^ Data are available under the terms of the Creative Commons Attribution 4.0 International license (CC-BY 4.0).

## Plain english summary

### Aims of the research

This study will increase understanding about the different ways in which end-of-life care is provided for children and young people, and examine how these different models of providing end-of-life care impact on children and their families.

### Background to the research

Around 4,500 babies, children and teenagers in England and Wales will require end-of-life care each year. Currently, the provision of this care varies across the country and little is known about how this variation impacts on children and their families. There are growing numbers of specialist palliative care services and children’s hospices in the UK, but there is little evidence to tell us how these services should be developed and what their role should be in supporting children and young people at the end of life. We also know very little about the costs of care and how best to use these resources to improve care for these children and their families.

### Design and methods

This research has three linked studies, which are termed workstreams in the overall study.

Workstream 1 (a survey) will identify the different models of providing end-of-life care for babies, children and young people (age 0–25) in neonatal units, paediatric intensive care units and cancer treatment centres.

Workstream 2 (a qualitative study) will learn more about these models by interviewing bereaved parents about their experiences of their child’s care at the end of their life, the impacts of this care, and how care could have been improved.

Workstream 3 (a quantitative study) will investigate the impacts (which we call outcomes) of the different models of end-of-life care for children and their families.

### Dissemination

We will work with parents, professional organisations and charities to disseminate the findings to inform future guidance and service development for end-of-life care for children.
