# Supplementary material for: End of life care for infants, children and young people (ENHANCE): Protocol for a mixed methods evaluation of current practice in the United Kingdom
Source: NIHR Open Res. 2022 May 13;2:37. [Version 1] doi: 10.3310/nihropenres.13273.1 (PMC7613236; doi:10.3310/nihropenres.13273.1)
Supplement: Peer review history

# Reviewer report: Reviewer response for version 1

**DOI:** 10.3310/nihropenres.14392.r28687

**Published:** 2022-07-04

**Authors:** Rafael Romo

**Affiliations:** 1 Dominican University of California, San Rafael, CA, USA

Thank you for the opportunity to review this protocol report for a mixed methods study to evaluate and understand end-of-life care models for children and their parents in the UK. 

 This is a well written report that provides a well-articulated justification for the study. The study is comprehensive, involving key stakeholders in the process, save for the children themselves (who may not be possible to include). The multi-pronged approach should provide excellent, detailed data on which to make important recommendations to policy and future research.

 I have one substantive critique to provide: I believe the authors should provide some justification for using the surprise question as an indicator of the need for end-of-life care. The original intent of the surprise questions was not to be a prognostic estimate ^1^, and its predictive value for prognosis has been shown to be poor ^2^. While asking providers to identify patients using the surprise question is a good starting point, it should be one component of an overall estimate, as is done with the Gold Standards Framework.

Is the study design appropriate for the research question?

Yes

Is the rationale for, and objectives of, the study clearly described?

Yes

Are sufficient details of the methods provided to allow replication by others?

Yes

Are the datasets clearly presented in a useable and accessible format?

Not applicable

Reviewer Expertise:

Adult end-of-life care, decision making, advance care planning

I confirm that I have read this submission and believe that I have an appropriate level of expertise to confirm that it is of an acceptable scientific standard.

## References

[1] : The utility and value of the “surprise question” for patients with serious illness. *CMAJ* .2017;189(33) : 10.1503/cmaj.733231 E1072-E1073 10.1503/cmaj.733231 Reference source PMC556660928827439

[2] : The “surprise question” for predicting death in seriously ill patients: a systematic review and meta-analysis. *CMAJ* .2017;189(13) : 10.1503/cmaj.160775 E484-E493 10.1503/cmaj.160775 Reference source PMC537850828385893

# Reviewer report: Reviewer response for version 1

**DOI:** 10.3310/nihropenres.14392.r28643

**Published:** 2022-06-20

**Authors:** Toni Wolff

**Affiliations:** 1 The Children's Development Centre, Nottingham Children’s Hospital, Nottingham, UK

This is a mixed methods study of the different models of provision of children's palliative care across the UK with evaluation of the experiences of staff, patients and their families and an assessment of outcomes, resources used and costs to health care providers. The results will be useful for future service planning.

 The rationale for, and objectives of the study are explained well in the introduction and are well referenced. 

 The design and methods are clearly described and appropriate for the study.

 Surveying clinical or palliative care leads in each of the tertiary paediatric intensive care units, neonatal units and children's cancer services is a good strategy to gather information on the different models of children's palliative care within those units and also in the various districts that they serve. The plan to involve a Parent Advisory Panel in the development of the survey content, and also the plan to pilot the survey before role out will be beneficial. Interviewing the children's palliative care regional network leads will add further information about the children's palliative care services in each region and the particular barriers faced in service development. 

 The experiences of bereaved parents who were supported in the different models of care will be ascertained by interview. It is important that the interviewers are professionals with experience in supporting parents of children with life limiting conditions and that there is an identified trusted health care professional to support each parent as stated in the protocol. 

 The protocol states that focus groups of staff working in each model will include staff working in different roles including nursing and therapy, from community and hospice services as well as hospital based services. This is essential to give a fuller view of the model.

 Involving a parent in the analysis of the data from surveys and interviews is a good plan to help to ensure authenticity. Would it be possible to involve one or several young people with life limiting conditions at some point in the analysis, to help identify and refine the key themes?

 The proposed measures of outcome and resource use are comprehensive and realistic.

Is the study design appropriate for the research question?

Yes

Is the rationale for, and objectives of, the study clearly described?

Yes

Are sufficient details of the methods provided to allow replication by others?

Yes

Are the datasets clearly presented in a useable and accessible format?

Not applicable

Reviewer Expertise:

Children's palliative and end of life care

I confirm that I have read this submission and believe that I have an appropriate level of expertise to confirm that it is of an acceptable scientific standard.
